# A Manifesto in Defense of Pain Complexity: A Critical Review of Essential Insights in Pain Neuroscience

**DOI:** 10.3390/jcm12227080

**Published:** 2023-11-14

**Authors:** Javier Picañol Párraga, Aida Castellanos

**Affiliations:** Laboratory of Neurophysiology, Biomedicine Department, Faculty of Medicine and Health Sciences, Institute of Neurosciences, University of Barcelona, 08036 Barcelona, Spain

**Keywords:** pain, chronic pain, nociception, sensory neuron, pain neuroscience education

## Abstract

Chronic pain has increasingly become a significant health challenge, not just as a symptomatic manifestation but also as a pathological condition with profound socioeconomic implications. Despite the expansion of medical interventions, the prevalence of chronic pain remains remarkably persistent, prompting a turn towards non-pharmacological treatments, such as therapeutic education, exercise, and cognitive-behavioral therapy. With the advent of cognitive neuroscience, pain is often presented as a primary output derived from the brain, aligning with Engel’s Biopsychosocial Model that views disease not solely from a biological perspective but also considering psychological and social factors. This paradigm shift brings forward potential misconceptions and over-simplifications. The current review delves into the intricacies of nociception and pain perception. It questions long-standing beliefs like the cerebral-centric view of pain, the forgotten role of the peripheral nervous system in pain chronification, misconceptions around central sensitization syndromes, the controversy about the existence of a dedicated pain neuromatrix, the consciousness of the pain experience, and the possible oversight of factors beyond the nervous system. In re-evaluating these aspects, the review emphasizes the critical need for understanding the complexity of pain, urging the scientific and clinical community to move beyond reductionist perspectives and consider the multifaceted nature of this phenomenon.

## 1. Introduction

Pain is the chief reason for emergency medical consultations [1]. Beyond this immediate concern, the escalating incidence of chronic pain in recent decades has emerged as a major health challenge. It is now recognized as a primary contributor to disability and work-related absenteeism, reflecting profound socioeconomic implications [2,3]. The taxonomy of chronic pain remains under scrutiny, especially regarding whether it should be considered merely as a symptomatic manifestation that lasts more than 3 months or acknowledged as a distinct pathological condition [4]. In fact, the category “Chronic Pain” is included in the ICD-11 (International Classification of Diseases) comprising seven different groups of chronic pain [5]. This distinction plays a pivotal role in shaping discussions around its therapeutic strategies and the necessity for patient-specific interventions.

In the realm of chronic pain management, in some conditions, a discernible paradox emerges: Despite the proliferation of medical interventions, the prevalence of pain disorders persists unmitigated [6]. Considering the modest outcomes associated with many pharmacological regimens and the recalcitrant nature of pain across an individual’s lifespan, there is an evident gravitation towards investigating non-pharmacological interventions that promise cost-effectiveness and notable improvements in patient quality of life [7,8].

Notably, contemporary clinical guidelines for diverse chronic pain conditions advocate for the integration of therapeutic education, structured physical activity, and cognitive-behavioral therapy as foundational therapeutic avenues [9,10,11]. Within this context, the past decade has witnessed an augmented focus on pain neuroscience education, conceived to equip patients with a coherent comprehension of their condition. This strategy seeks to dispel prevailing uncertainties, curtail fear-avoidance tendencies, and fortify patient self-efficacy, among others [12].

With advancements in cognitive neuroscience, there emerges an interpretation that posits pain primarily as a brain-derived output. In a figurative context, pain pedagogy underscores pain as a cerebral appraisal [13]. This viewpoint aligns with the tenets of Engel’s Biopsychosocial Model, positing that disease manifestation is not merely a consequence of biological underpinnings but is intricately interwoven with psychological and social factors [14].

This shift in paradigm has introduced nuanced complexities, potentially leading to misconceptions that bear substantial ramifications for patients, educators, and researchers in practical settings. Consequently, within this review, we endeavor to re-examine the intricate mechanisms underpinning pain, emphasizing the necessity to eschew reductionist interpretations of this multifaceted phenomenon.

Highlighting the seminal findings of recent years that challenge long-held assumptions in both clinical and research domains: Is pain experience a cerebral phenomenon? Have we conflated nociception with pain? Is the chronification of pain solely attributable to central mechanisms? Have we misunderstood central sensitization syndromes? Is there a specific neuromatrix dedicated to pain processing? Have we overlooked critical elements beyond the nervous system?

## 2. Pain and Brain: A Mereological Fallacy We Should Abandon

The nascent period of neuroscience and the exploration of cortical studies engendered certain “cerebrocentric” tendencies. In this paradigm, the brain was posited as the sole agent and thus the focal point of pain. This notion pervaded the pedagogy of pain, forging viewpoints profoundly shaped by the currents of functionalism and connectionism. This led to the propagation of aphorisms such as “pain is an output of the brain” or “pain is an opinion of the brain” [13]. A conception that emerged from connectionist analogies describes that the brain processes information through neural networks, similar to how a computer would use its circuitry to perform computations. Continuing with this, the brain would take stimuli as inputs, producing outputs in the form of behaviors and perceptions [15]. This all culminates with the hypothesis by several authors that when the brain perceives a threat (or potential threat), it decides to produce pain for the protection of the organism. Where “the impact of pain is dependent on the value of the perceived threat” [16], rescuing an “evaluativist” vision of pain [17].

It is true that this perspective may be backed by some evidence, where several authors uphold this connectionist hypothesis when facing a threat. For example, connectivity prior to a stimulus can modulate and determine the perception of pain [18], dopamine and the reward system (relevant in motivational states) influence the perception of pain [19], the insula plays an important role in the chronification of pain [20], complex emotions such as nostalgia modulate the perception of pain through thalamocortical mechanisms [21], and brain activity may monitor and modulate the relevance and degree of pain [22], among a host of other studies focused on brain research in pain contexts through neuroimaging. However, are these data examples truly supportive of the mentioned narrative?

We must be cautious in how this evidence is interpreted and avoid falling into excessively “brain-centric” reasoning, as this reduces a complex human and subjective experience to merely the consequence of an organ, thereby falling into the known mereological fallacy [23]. A concept that describes the error of attributing characteristics and subjective experiences to single components of a whole. In fact, some authors highlight this fallacy as “the central error of many cognitive neuroscientists” [24]. Pain is more understandable when we assign it to the whole itself and to the emergent properties that arise from the interactions of the components of a complex system. This is not limited to pain but applies to biology itself, also viewed epistemologically from the perspective of complex systems [25,26]. Ascribing aspects solely to the brain, disregarding the organism, constitutes a fallacy, falling into speculation rather than empirical evidence and fact-checking.

Building upon this discourse, we have now come to recognize that even pain typically ascribed to central mechanisms often embodies alterations or involvement within peripheral systems. Consider, for example, phantom limb pain—an aftermath of amputation—which has historically been perceived through a predominantly brain-centered lens, attributing this condition to cortical reorganization [27]. However, the significant role that peripheral factors play in both the onset and maintenance of this pain has often been insufficiently acknowledged [28]. These consist of the interplay of peripheral neuromas, the influence of ectopic discharges, and the sensitization of dorsal root ganglia, among others [29]. In fact, interventions aimed at the periphery can also yield effective results in mitigating phantom limb pain [30]. Therefore, the binary discourse debating whether phantom limb pain is a bottom-up or top-down process is not as straightforward, and the pivotal contribution of peripheral mechanisms to changes in cortical reorganization, among other factors, should not be underestimated.

Fibromyalgia presents another salient example of a condition characterized by widespread chronic pain. Owing to its apparent nonspecific nature, it has over recent decades been relegated to a controversial catch-all category, laden with labels and stigma, where the condition seemed reduced to “psychosomatization”. This perspective perhaps still persists today in Western societies [31]. However, fibromyalgia is now more widely recognized, and neuroimaging studies have revealed significant alterations in the brain areas that have been identified as key regions in the pain experience, as described by Melzack. Along with the popularization of the concept of central sensitization, this led to a portrayal of fibromyalgia as a condition of purported “pain amplification” and hypersensitivity, where, once again, the brain ultimately decides to elicit it [32]. These terms and conceptions continue to provoke rigorous analysis and debate in the field today. Moreover, this point of view inadvertently ignores the other side of the story. If we consider peripheralist perspectives, it is known that the peripheral nervous system can play an essential role in the production and maintenance of pain [33]. For example, there is a potential link between fibromyalgia and pathological changes in small nerve fibers, characterized by a reduction in the density of both myelinated and unmyelinated fibers [34], low-grade systemic inflammation which can affect nociception itself [35,36,37], changes in gut microbiota [38], and microvascular alterations, among other factors [39].

These examples of two contexts commonly attributed to cortical reorganization cast doubt on the occasional reductionist tendency within the field of pain. However, it is not solely a matter of the peripheral nervous system interacting with the central system but also the myriad contributors that continuously modulate its activity. This underscores the importance of other factors such as the immune system and hormonal variables [40].

In this context, it is imperative to approach dichotomous perspectives with caution. Within the biomedical domain, we must circumvent the fundamental missteps that have historically ensnared other disorders, as exemplified by the serotonergic hypotheses associated with depression [41]. In our area of study, adherence to simplistic metaphors, such as an imbalance between excitation and inhibition, may lead to an oversimplified belief that medical intervention targeting one pathway could suffice to ameliorate the condition. Nonetheless, the contentious application of opioids and antidepressants, accompanied by their long-term effects, is an example that reflects a nuanced complexity that transcends any binary categorization [42,43,44]. The current predicament surpasses the realm of specific pharmaceuticals, presenting us with a debatable issue of healthcare medicalization and/or over-medicalization that also demands critical scrutiny [45]. Concurrently, the burgeoning trends in cognitive-behavioral modalities must not neglect the vast expanse of biomedical knowledge, acknowledging that the experience of pain transcends mere cerebral interpretation, a perspective that has to consider the under-treatment of pain that is present in some contexts [46].

Ultimately, caution is necessary to avoid the mereological fallacy in the field of pain, where hypotheses postulating that the perceived threat compels the brain to produce pain are, to some extent, unfalsifiable; hence, many of them are unscientific. Such perspectives should not detract from the body of knowledge that has been accrued in the field of pain science today.

## 3. Discerning the Ambiguity between Pain and Nociception: A Crucial Source of Misnomers

The International Association for the Study of Pain (IASP) diligently examines its ontology and epistemology, meticulously crafting logical terms and definitions published by the association [47]. However, when confronted with terms not adequately defined by the IASP, a scenario of epistemic gaps emerges, potentially leading to confusion [48]. In this light, pain neuroscience education has gained prominence as a prevalent clinical approach in recent years [49]. Within such a framework, statements such as “pain is not nociception” or “pain is not tissue damage” have been frequently reiterated. Nevertheless, the amalgamation of inherent simplicity and continual repetition may instigate both the bias of plausible simplicity and the availability bias, where simplified and repetitively echoed statements become accepted truths without question [50,51]. These biases can significantly influence decision-making processes not just in clinical settings but also within scientific contexts [52]. 

This sets the stage where these assertions run the risk of generating confusion and misinterpretations. As a result, we must confront an essential question: Are we over-simplifying these distinctions to the point where we risk creating fallacies?

On the one hand, the International Association for the Study of Pain (IASP) acknowledges the inherent complexity that characterizes pain, defining it as an unpleasant sensory and emotional experience associated with actual or potential tissue damage, or described in terms of such damage [47]. Therefore, pain is connected to a highly individual experience, characterized by unique properties shaped by qualia [53]. This concept, which has been popularized in the fields of philosophy of science and mind, encapsulates the subjective and idiosyncratic qualities of perception. It highlights the intricacy involved in comprehending pain, echoing the so-called hard problem of consciousness: The enigma of how, why, and at what point a subjective experience originates from physical processes within our bodies [54].

This phenomenon is also inseparable from the exploration of the interrelation between mental phenomena and their substrates [55]. Hence, pain as a qualia cannot merely be reduced to nociception but rather requires considering the subject’s subjective experience with the accompanying high degree of interindividual variability.

Nociception, on the other hand, is conceived as the neural substrate linked to harmful or potentially harmful elements. This term is specifically defined by the IASP as the neural process of encoding noxious stimuli [56]. Despite the clarity of the primary distinction between the terms—pain and nociception—a plethora of misinterpreted concepts persist. It must be remembered that pain is not a “concrete thing”, and the representation of it is heavily influenced by language [48]. In this context, countless misnomers are encountered at both the scientific and clinical levels: Names or terms applied incorrectly that ultimately lead both research and clinical practice astray [57]. Notable misnomers include concepts like pain thresholds, pain processing, pain amplification, pain fibers or pathways, and pain hypersensitivity, among others [48]. Rather than providing clarification, these concepts contribute to confusion in the field of pain-related medical education.

In addressing this topic, a substantial part of pain pedagogy leans towards reductionism, often resorting to thought experiments and contentious, debatable case studies. One such case involves a situation where a man presented to the emergency room with severe pain despite the absence of any apparent noxious stimulus [58]. The case recounts an incident where a man suffered an accident at work involving a large nail that fell onto his foot. This accident caused him intense pain, but upon removing his boot, the nail was found lodged between his toes without any evident tissue damage. This scenario prompts the question: Does it genuinely illustrate the existence of pain without nociception and/or damage?

The reality remains that the evidence supporting this assertion is far from compelling, and these perspectives run the risk, erroneously, of leading patients and clinicians back to a psychogenic phenomenon. Untested thought experiments and anecdotal evidence distance us from a reality that we must keep in mind: Nociceptive activity remains one of the strongest predictors of the experience of pain [59,60]. Demonstrating the empirical manifestation of pain without underlying nociceptive activity is truly challenging. It is crucial to maintain a critical stance and rethink new propositions that have emerged in the past decade, such as pain without nociception. Once again, this assumption deviates from evidence-based substantiation and should be approached with caution, at least for now.

However, it is true that the relationship between nociception and the perception of pain is not linear. Nociceptive information is modulated by a variety of factors. In this context, cognitive factors, such as attentional processes, expectations, and placebo, can greatly influence the final perception of pain [61]. Emotional states [62], uncertainty [63], descending inhibitory and facilitatory modulation [64], and other sensory inputs, as proposed by the Gate Control Theory [65], are among the many factors that can also play an important role.

Within this context, specifically concerning therapeutic pain education, a debate may arise between the utility and accuracy of language [66]. We find ourselves coexisting with concepts we have had to abandon in recent years despite their seeming usefulness, precisely because of the inherent nocebo effect they carry. In an effort to improve pain management, new conceptualizations that encompass advancements in the understanding of pain were proposed. However, in agreement with Cohen et al., language is all we have in some respects, and utility should not overshadow the precision required by the complexity of pain—particularly considering that pain may not be a “thing” with inherently active properties or characteristics [67].

Hence, all of these aspects bring nuance to the traditional notions we hold, indicating the inherent complexity of both nociception and pain. While these concepts are not synonymous, they are not so distinct as to be separated by a clear-cut line in pain pedagogy. Moreover, it is crucial to acknowledge the profound importance of language, as it plays a guiding role in shaping our understanding.

## 4. Starting from the Periphery: Does Pain Modulation Begin in the Central Nervous System?

From a reductionist standpoint, it can be observed that the study of pain has encompassed both cerebrocentric and peripheralist perspectives. Initially, the understanding in this field was markedly shaped by the Cartesian viewpoint, a paradigm that propelled a significant leap forward in the physiology of pain by Descartes proposing that pain emanated from nociceptive projections directed towards the pineal gland [68]. As time progressed, the focal point of study gradually transitioned towards the central nervous system.

At present, it is acknowledged with considerable certainty that noxious (or potentially noxious) stimuli, whether originating internally or externally, are transduced to nerve impulses. This transduction is facilitated largely due to the pivotal role of nociceptors and some mechanoreceptors, which are endowed with a vast array of ion channels specialized in sensing and reacting to various environmental factors, such as temperature, mechanical stress, or pro-inflammatory conditions, among numerous others [69]. 

Within this complex system, there are ion channels vital for nociception: those that facilitate the transduction of specific stimuli through the influx of calcium and/or sodium (TRP family, P2X, ASIC, PIEZO, etc.), voltage-dependent channels that hold significant relevance in the genesis and propagation of action potentials (Nav, Cav, etc.), and channels that govern potassium discharge (Kv, Kir, K2P, KaCa, KNa, etc.), among others (5-HT3, HCN channels, TMEM16, TKr, etc.) [70]. Furthermore, these nociceptors are essentially pseudounipolar neurons, with their somas situated within the dorsal root ganglia, a structure integral to nociception. However, it must be emphasized that the scenario is more complex than it seems, and based on their individual characteristics, various types of nociceptors can be identified.

Based on myelination levels, most nociceptors are categorized as C fibers, which have small diameters and low myelination, conducting impulses at speeds between 0.4 and 1.4 m/s. In contrast, A fibers feature higher myelination and faster conduction speeds ranging from 5 to 30 m/s [71]. However, it is clear that the complexity goes beyond mere myelination levels and is also determined by their specialized roles, governed by differences in expression patterns. Within this context, several nociceptors have been identified, each exhibiting different sensitivities and functionalities. For instance, there are non-peptidergic mechanonociceptors that seem to respond solely to mechanical stimuli (MrgprD^+^), alongside peptidergic nociceptors sensitive to harmful cold temperatures (TRPM8^+^). Furthermore, some peptidergic nociceptors are responsive to both noxious heat and, likely, mechanical stimuli, identified by markers, such as TRPV1, TRKA, and CGRP. Additionally, A fiber mechanoreceptors without free nerve endings play a crucial role in mechanonociception. These include receptors mediating pin-prick pain, characterized by TRKA, CGRP, and Npy2r presence, as well as those facilitating painful mechanical sensitivity (TRKA and CGRP^+^) [72]. However, this classification is complex, encompassing silent nociceptors that become active in the presence of inflammation [73], as well as low-threshold C fibers serving as mechanoreceptors, facilitating the pleasant touch [74].

In light of the information discussed, the activity of nociceptors is intrinsically intertwined with the immune system’s operations. This correlation is prominently illustrated through the events of peripheral sensitization, a condition delineated by the IASP as a state of “Increased responsiveness and diminished threshold of nociceptive neurons in the periphery to the stimulation of their receptive fields” [56]. In an effort to elucidate the neurobiological mechanics of this phenomenon, previous investigations have revealed a plethora of mediators in the extracellular milieu, alongside a sophisticated cross-talk with the immune system. This interaction is capable of triggering signaling cascades that ultimately augment the sensitization of nociceptors, enhancing their responsiveness to external and internal stimuli [75].

Beyond the essential characterization of peripheral phenomena and peripheral sensitization, there are recent and significant implications that should not be overlooked in this field. Indeed, in a popularized and simplified manner, it has been established that the integration of information and modulation of nociception commence at the spinal cord level, grounded on the Gate Control Theory, which hinges on the action of inhibitory interneurons [65]. This assumption might be steered by the apparent absence of inhibitory interneurons and synapses in the periphery.

However, recent propositions suggest that intrinsic GABAergic signaling is in operation within the dorsal root ganglion (DRG) itself, potentially serving as the “first peripheral gate” at the axonal bifurcation of the DRGs [76,77]. The discourse extends beyond merely addressing GABAergic modulation in the DRGs; it additionally underscores the importance of the peripheral opioid and endocannabinoid systems [78,79]. This includes their active roles at the terminals, where they appear to mediate a substantial portion of the analgesic effects of synthetic cannabinoids, notably through the engagement of CB1 receptors [80]. Furthermore, observations point to a diminished release of inflammatory mediators in states of peripheral sensitization, a process mediated through CB2 receptors in the immune system [81]. This delineates an area of research that demands further intricate exploration and deciphering.

Furthermore, a frequently neglected facet regarding the significance of the periphery resides in the inherent spontaneous activity of sensory afferents, which might be intricately linked with a sustained feedback interaction with the central nervous system (CNS), fostering a persistent somatosensory alteration. Indeed, this peripheral activity could play a pivotal role in sustaining central sensitization [82]. Despite the prevailing assumption that pain is perpetuated by central mechanisms in pathophysiological contexts, emerging evidence suggests that peripheral hyperexcitability and spontaneous activity might be intricately connected to pain [83,84]. These occurrences are closely associated with membrane potential instabilities (MPIs), manifesting as membrane potential oscillations or spontaneous depolarizing fluctuations, potentially serving as a theoretical model for ectopic discharges and repetitive firing, among other phenomena [85]. It seems that both MPIs and spontaneous activity might be correlated with the spontaneous opening of channels permeable to Na^+^ and/or Ca^2+^ [83,84,85,86,87].

Additionally, recent findings illuminate the complexity of peripheral cross-talk, including the transfer of mitochondria from macrophages to nociceptors as a modulation strategy for inflammation [88], the role of cytokines such as the macrophage migration inhibitory factor in the previously mentioned spontaneous activity [89], the critical communication between satellite glial cells and the DRGs [90], and the regulation of nociceptor sensitization through top-down mechanisms, including the engagement of the HPA axis in the peripheral nociceptors regulation [91].

Therefore, we encounter evidence that underscores pivotal aspects in understanding nociception, which ought to be emphasized: (1) the inherent dynamic adaptability of the peripheral nervous system to transition between various states, (2) the ongoing discourse regarding whether the subjective perception of different experience of pain stems from the processing and integration of all sensory inputs or, alternatively, from the specific neural activity of various subtypes of sensory afferents, (3) the critical role of continuous communication between the immune system and the nervous system operating as a cohesive unit, (4) the chronification of pain is not solely rooted in alterations within the central nervous system, and (5) both the integration and modulation of nociception are ubiquitous phenomena. Consequently, this perspective challenges a cerebrocentric view of pain emergence, wherein the significance of the peripheral contributions cannot be dismissed.

## 5. Central Sensitization as a Focus of Confusion: Weaving Threads of Uncertainty

The spinal cord plays a pivotal role in transmitting and processing information. Peripheral nociceptors send information to the dorsal horn, where second-order neurons receive and transmit it in an ascending manner to cortical areas. However, within the dorsal horn, a complex network of excitatory and inhibitory interneurons utilizes glutamate and GABA (among others) as key neurotransmitters to modulate the transmission of nociceptive signals, ensuring precise and accurate modulation [92]. Additionally, descending projections from higher brain regions, including the periaqueductal gray matter, rostroventral medulla, dorsal reticular nucleus, and ventrolateral medulla, exert regulatory control over nociception. These descending pathways critically modulate nociception, contributing to the overall pain experience [93].

The synapses formed between nociceptive afferents and second-order ascending neurons are located in key regions of the dorsal horn. In fact, Rexed classified spinal cord neurons into different laminae (I–X) based on their size, shape, and structure [94]. Specifically, laminae I, II, and III play a crucial role in the processing of nociceptive information, receiving inputs from unmyelinated polymodal C fibers and thinly myelinated Aδ fibers [95].

Within these laminae, intricate neural circuits are formed, where components of the posterior horn are interconnected with multiple interneurons and primary afferents. The proposal of the Gate Control Theory by Melzack and Wall emphasized the importance of these circuits [65]. In this aspect, injury or inflammation, for instance, can lead to the development of hyperalgesia, allodynia, and spontaneous pain. These processes are believed to involve changes at the level of these dorsal horn synapses, including synaptic plasticity (long-term potentiation, LTP), reduction in inhibitory GABAergic/glycinergic neurotransmission, and alterations in the properties of mechanoreceptive afferents, among other mechanisms [96]. Therefore, this area is essential for understanding nociception.

Along the same line of inquiry, it was Woolf who conceptualized and characterized the phenomenon of central sensitization in preclinical studies. This intricate phenomenon involves enduring modifications in the excitability of second-order neurons within the spinal cord, elicited by heightened afferent activity, thereby intricately altering the somatosensory system itself [97]. The profound implications of this concept were further underscored by the seminal discovery of long-term potentiation (LTP) in the hippocampus by Bliss and Lomo, where synchronous high-frequency input was found to engender synaptic efficacy enhancement [98]. Subsequently, analogous mechanisms were unearthed in the spinal cord with the discovery of long-term depression (LTD) [99].

Today, LTP is recognized as an indispensable mechanism underpinning our comprehension of central sensitization [100]. The substrates underlying this synaptic plasticity are profoundly activity-dependent, intricately governed by glutamatergic neurotransmission and the modulation of post-synaptic AMPA and NMDA receptors, among other factors. Indeed, the neurobiological underpinnings of central sensitization extend far beyond mere adjustments in synaptic efficacy (particularly those confined to activity-dependent modifications). They encompass comprehensive transformations in neural circuitry, manifested by an augmented release of neurotransmitters from the presynaptic neuron, a down-regulation in inhibitory signaling, modulations in membrane excitability, adaptations in microglial responsiveness, and astrocytic disturbances, among a multitude of other nuanced alterations.

The progressive comprehension of central sensitization has emphatically underscored the clinical significance and pragmatic repercussions of these biological underpinnings in the realm of pain perception, a clinical viewpoint defended by Woolf [101]. Concurrently, Yunus, within the context of clinical research, pioneered and substantiated the perspective that various diffuse clinical presentations (such as fibromyalgia, myofascial pain syndrome, chronic fatigue, and irritable bowel syndrome, among others) exhibited considerable commonalities in the absence of discernable tissue origin. This observation prompted him to introduce the concept of “central sensitization syndromes” (CSS), constructing a theoretical model that was predicated on the phenomenon of “central sensitization” [102]. Nevertheless, the association drawn between central sensitization and CSS was an extrapolation from deductive reasoning. In fact, it was postulated that the atypical responses of subjects to thermal stimuli (among others), coupled with discernable neuroimaging alterations in reaction to these stimuli, indirectly hinted at the central sensitization mechanisms proposed by Woolf.

Abiding by the foundational principles of logic, the presented argument does not withstand a valid or scientifically robust line of reasoning. It reveals a potential flaw in deduction, where strong general premises based on preclinical studies with animal models (central sensitization) lead to specific conclusions in another distinct area (clinical presentations). Within this deductive framework, there can be an occasional failure to contemplate all potential models when addressing a problem.

Thereby leading to the inference of conclusions that remain in the realm of possibility rather than being definitively correct or accurate [103]. Ergo, the application and tacit acceptance of these premises (central sensitization as the main mechanism of CSS) remain notably contentious. In fact, at present, we lack a definitive method to establish and demonstrate the presence of central sensitization in human subjects. 

Moreover, recent systematic reviews suggest that purported central sensitization questionnaires do not entirely align with sensory aspects. However, they demonstrate a substantial correlation with psychological constructs, such as depression, catastrophizing, anxiety, stress, and kinesiophobia, among others [104]. Furthermore, other reviews that incorporate neuroimaging studies from diverse pathological contexts fail to support the concept of CSS. In these studies, it is observed that, notwithstanding a commonly amplified response to stimuli, the assorted phenotypes remain indistinguishable in their classification. This underlines that marked heterogeneity is observed in individual differences, both across different syndromes and within the same syndrome [105].

Notwithstanding the aforementioned, over the course of time, the hypothesis that central sensitization underlies chronic pain has surreptitiously transitioned into an assumption. This shift seems to dismiss the fact that central sensitization has been delineated within laboratory environments, employing electrophysiological experiments that facilitate the recording of neural activity through a range of paradigms.

Thus, the mystified concept of centralized pain resulting from central sensitization became ingrained in clinical practice, research, and education [106,107].

In a similar vein, these interpretations and classifications gained traction within the field of pediatrics, encapsulating constructs such as amplified pain syndrome. This sweeping categorization includes non-specific headaches, generalized musculoskeletal pain, and various types of abdominal discomfort, among others. All of these conditions exhibit a hypothetic unifying feature: central sensitization [108]. These challenges reemerge due to persistent epistemological confusion, where the purported hypersensitivity integral to central sensitization is not directly attributed to pain itself. Instead, it could arguably and speculatively (at least in humans) refer to phenomena that modulate nociception, ultimately influencing the painful experience. Despite the latter, these pathophysiological scenarios continue to be perceived as manifestations of central sensitization, thereby inducing hypersensitivity. This leads us to a circular argument: “The primary pathophysiological feature is a sensitized central nervous system that results in an enhancement in the processing of pain and sensory stimuli” [107,108]. Upon close scrutiny, it seems that the pain is ascribed to sensitization that heightens hyperexcitability, consequently intensifying pain—an argument that paradoxically uses itself to explain its premise [108].

However, these presuppositions pertaining to central sensitization have found broad acceptance in the domain of clinical taxonomy, thus endorsing premises that are currently inaccurate. The objective of this review is not to dispute the potential role of central sensitization as a mechanism underpinning chronic pain states; rather, it challenges the notion of CS serving as the primary etiopathogenesis in an array of contexts. Concurrently, it underscores the necessity for a discerning stance on generalizations—a fundamental pitfall that needs to be rectified in scientific endeavors. Just as the recourse to circular arguments for elucidating intricate pathologies should be avoided. At this juncture, it is incumbent upon us to critically examine our inferences and adopt more appropriate provisional concepts, while exploring and corroborating the factors substantiated in pathologies of such profound complexity. In the same context, it is vital to acknowledge that central sensitization, as characterized in animal models, is not merely a process of synaptic plasticity. Indeed, its neurobiological underpinnings extend to encompass the neural circuitry at large [101].

## 6. Cortical Processing: Does the Pain Neuromatrix Really Exist? A Controversial Simplification

The information derived from nociceptors is conveyed to higher cortical areas through various ascending pathways within the anterolateral system, facilitating its processing through the coordinated interaction of distinct brain regions. This system encompasses the spinothalamic tract, spinoreticular tract, and spinotectal tract and was evidenced by previous investigations utilizing post-mortem and neurosurgical studies, which also proposed a discernible functional division within the spinothalamic tract [109]. Wherein a lateral and medial pathway exists, exhibiting differential transmission and processing of nociceptive information [110,111].

Within this framework, the lateral system has traditionally been recognized as a principal contributor to the sensory-discriminative aspects of pain, encompassing aspects such as localization, intensity, and duration [112]. This system involves the lateral thalamic nuclei, somatosensory cortices S1 and S2, as well as the insular cortex, which collectively contribute to the integration of sensory afferences [109]. Conversely, the medial system has been proposed as a comparatively slower pathway responsible for the processing of affective components of pain [112]. It encompasses structures like the medial thalamic nuclei, anterior cingulate cortex, prefrontal cortex, and key structures like the amygdala [113].

Clinical cases and lesion paradigms provide support for distinct functional roles of these areas in relation to pain, as evidenced by how localized lesions in the anterior cingulate cortex (ACC) induce alterations in affective pain components [114], while lesions in somatosensory areas impact the sensory-discriminative components of pain [115,116]. A particular instance exemplifying this role differentiation is pain asymbolia, a specific type of depersonalization. In such cases, individuals experience a dissociation wherein the pain perceived is not acknowledged as their own [117]. Such dissociative cases present two opposite poles: a division between the sensory facets of pain and the affective-emotional constituents, and vice versa [118].

In this aspect, it is important to clarify a fundamental distinction between essential percepts: pain and suffering as closely interconnected entities, albeit not synonymous [119]. Suffering can occur independently of pain, just as pain can be experienced without implicit suffering. These phenomena likely differ in their underlying biological correlates, which is crucial for understanding pain. In this aspect, pain is considered an acute stressor [120], and prolonged pain can lead to alterations in the correlates of pain unpleasantness, analogous to the effects of chronic stress [121,122]. For instance, studies have demonstrated that in healthy individuals, pain-related brain activity involves the ACC and anterior insula [123]. Conversely, chronic pain appears to engage cognitive areas of the insula and other corticolimbic regions associated with emotional processing [124,125]. Paradoxically, suffering can modulate our susceptibility to pain perception, with psychosocial factors such as catastrophizing, helplessness, and excessive rumination influencing pain experiences [126,127].

The growing availability of neuroscientific techniques like fMRI, PET, SPECT, and EEG, among others, has shed light on the significant role of cortical processing in pain perception. Former perspectives based on localizationism suggested the existence of specific and specialized regions responsible for pain perception [128], proposing the notion of a “pain center”. However, contemporary evidence suggests that pain does not stem from the activation of a single center but involves the coordinated engagement of multiple brain structures. This led to the hypothesis of a “pain neuromatrix”, a network of specific structures responsible for processing nociceptive information and generating the experience of pain [129].

However, this concept was originated by Melzack, who initially proposed the existence of a neuromatrix consisting of widespread neural networks that include somatosensory, limbic, and thalamocortical components. Together, these components contribute to the sensory-discriminative, affective-motivational, and cognitive-evaluative aspects of the pain experience [130]. Moreover, Melzack’s theory introduced the concept of a neurosignature, suggesting that pain is determined by the synaptic architecture of the neuromatrix, influenced by both genetic and sensory factors [130]. Importantly, the neuromatrix theory of Melzack was not limited to a specialized network solely dedicated to pain processing but plays a crucial role in perceptual outputs.

Many other researchers have further developed and characterized this theoretical model, leading to the emergence of the pain neuromatrix [131]. This term arose due to the observed correlation between perceived pain intensity and the magnitude of response within the structures of the pain neuromatrix [132]. Additionally, the pain neuromatrix areas demonstrate modulatory capabilities influenced by those factors that can reduce pain perception [133]. Consequently, the concept of a pain-processing network gained momentum in pain research, and some authors even consider the pain neuromatrix as a potential biomarker and an objective measure of pain perception [134,135]. However, these perspectives suggesting that pain is the exclusive percept emerging from this network have generated debate [136]. Therefore, it remains crucial to question whether those suggesting specific activation of the pain neuromatrix have robust experimental evidence to support their claims.

Looking at the other side of the argument, there are observations that challenge this model [129,131,136,137]. A significant portion of neural activity within the pain neuromatrix in response to nociceptive stimuli appears to be nonspecific to nociception itself [129]. Notably, certain points emerge: (i) no primary cortical area has been exclusively identified to process thalamocortical nociceptive input; (ii) no cortical column exhibiting preferential response to nociceptive stimuli has been described; (iii) specific nociceptive neurons have been reported across different areas, but their distribution is extensive, and their characterization is based on a high activation threshold. It is noteworthy that many of these neurons can also be activated by other sensory modalities, such as visual stimuli perceived as “threatening”; (iv) the relationship between perceived pain intensity and neuromatrix activation is nonlinear; and (v) the influence of context and novelty on neuromatrix activation has been emphasized [129,136]. These findings challenge the notion of specificity in exclusively eliciting pain. In fact, authors like Patrick Wall suggest that continuing to search for such specific cells is an act of faith [138]. Thus, it raises doubts about whether the information processed within these areas is intrinsically linked to pain or rather to the salience and relevance of sensory stimuli. It is plausible that some nociceptive projections may be involved in predicting and evaluating consequences, indicating a nonspecific role in pain perception.

The preceding discussion prompts the consideration of theoretical models that challenge the notion of a specific neuromatrix for pain. From an alternative perspective, the emphasis shifts towards the importance of the matrix in detecting the salience of sensory stimuli [136]. This view aligns with the nonlinearity often observed in the experience of pain, and the “pain neuromatrix” is conceptualized as a system involved in the detection, attentional orientation, and response to highly relevant sensory events within a specific context. This cortical system is believed to play a fundamental role in detecting events that are significant for bodily integrity, regardless of the sensory modality involved. Additionally, it is proposed that this system contributes to the construction of a multimodal cortical representation of the body and its immediate spatial surroundings, serving as a potential defensive system for the organism [136].

This salience hypothesis aligns with insights provided by other researchers in understanding the underlying mechanisms of reward and aversiveness at the cortical level, offering a more comprehensive perspective on the dynamic interplay between these constructs [139].

The unpleasantness of pain constitutes a crucial component in its understanding, and there is evidence to suggest that complex conditions such as chronic pain may share neurobiological foundations with addictive disorders [140]. Within this framework, functional magnetic resonance imaging studies have indicated that the impact of a reward diminishes in the presence of a threat, and conversely, the perception of a threat is attenuated in the presence of a reward. These findings support the notion that reward and threat processing are not inherently independent but rather engage in a competitive process. Key structures, including the anterior insula, ventral tegmental area, putamen, and striatum, are implicated in detecting and evaluating salient stimuli. This competitive system is hypothesized to enable the identification of stimuli crucial for the organism’s survival and adaptation [139].

Nonetheless, these models could overlook the unceasing predictive ability of complex organisms, a vital factor for survival. Recent developments in the computational realm of cognitive neuroscience and machine learning are introducing pain as a heuristic and probabilistic mechanism [141,142]. Indeed, the incorporation of pain probabilistic mechanism paves the way for effective maneuvering in a world saturated with uncertainty, a concept phenomenologically defined as “A state where a given depiction of the world cannot be employed as a compass for guiding subsequent behavior, cognitive processing, or emotional response” [143].

It is therefore noteworthy to emphasize the emergence of a Bayesian approach to comprehending pain [144]. In fact, perception itself follows a probabilistic model to some extent, allowing for the management of ambiguity and the filling of informational gaps with prior knowledge. Thus, the perception of pain extends beyond the mere processing of sensory information, incorporating predictions based on past learning experiences. In fact, the concept of chronic pain has been described by some researchers as aberrant Bayesian inferences [145], highlighting the role of predictive processes in shaping the experience of ongoing pain. Within this context, it is essential to recognize the fundamental role of learning processes in the understanding of pain. Evidence suggests that both classical and operant conditioning mechanisms significantly contribute to the complex phenomenon of pain [146,147,148]. Moreover, studies have demonstrated the capacity to evoke nocifensive behaviors by exploring specific engrams, which represent the intricate configuration of neural connections associated with a particular memory [149]. These learning processes also modulate nocebo and placebo effects arising in pain treatment through expectations, where prior conditioning and/or suggestion can influence both the exacerbation and alleviation of the patient’s pain experience [150,151,152].

Referencing the Bayesian Model within the context of pain indirectly references one of many modern theories of consciousness (ToC), specifically the comprehension of consciousness through predictive processing [115,153]. This approach suggests a framework for the systematic mapping of neural mechanisms onto certain domains of consciousness. Within this context, key dichotomies must be noted when discussing different theories of consciousness [154]. These include (i) global states versus local states, where the former is understood as levels of consciousness and the latter correlates with conscious contents or qualia; (ii) phenomenological properties versus functional properties, each having a unique objective; and (iii) the selection of a local state (why a subject possesses a specific local state) versus the experiential characterization of local states (why a specific local state is tied to a particular experience).

In essence, various theories of consciousness align with one or some of these perspectives, yet there remains a lack of a single, comprehensive theoretical model that satisfactorily accounts for consciousness in its broadest form. Regardless, these theories are crucial for pain understanding. Some of them emphasize higher-order processing (High-order theories) [155], while others focus on a physical substrate capable of emulating a virtual workspace (Global-workspace theories) [156].

There are theories that underscore mathematical quantification, depicting the degree of information produced by a collection of elements and its irreducibility to the information generated by its constituent parts (Integrated-information Theory) [157], or the ones related to top-down processing, such as predictive theories [153] or re-entry theories, which suggest that information does not solely flow in one direction, from sensory areas to more complex processing areas. Instead, they propose a reverse information flow from higher to lower regions, serving as an essential re-entry in consciousness and perception [158].

Even though these represent different viewpoints, they typically share a common factor leading to a focus on a concept often mentioned but rarely delved into deeply: “information”. Numerous hypotheses are framed in terms of information and abstraction, and this gives rise to a question: What is this entity termed as information?

While information is intangible, it remains a constant presence in organisms, ranging from the simplest to the most intricate levels. In the realm of neuroscience, information is conceptualized as a dynamic process encompassing the encoding, transmission, and decoding of diverse and innumerable neural activity patterns. This understanding is grounded in the application of the Information Theory, a theoretical backbone for many contemporary scientific disciplines that centers around the mathematical quantification of information [159,160]. This theory serves as one of the most reliable pathways for deciphering the neural code and stems from the principle of applied probability in information transmission within communication systems. It determines the distribution of possible outputs according to specific signals [161]. However, the implementation of Information Theory within neuroscience poses significant complexity. Neural modeling must not only consider the stimulus but also all preceding states. In this context, a stimulus can be depicted as a vector of various parameters, each symbolizing a preceding state of the stimulus that is pertinent to the response under scrutiny. For instance, in the case of a stimulus capable of taking on eight different values and a response contingent upon seven previous states, there would emerge 16,777,216 (8^7) distinct stimulus conditions [162]. Thus, it is evident that the application of Information Theory carries immense value for understanding Neuroscience, and by extension, the nature of pain.

In this aspect, it is critical to acknowledge the significant heterogeneity inherent in the neural correlates of the pain experience and the overarching abstract concept of information, which presents challenges in terms of external validity and the potential for generalization within scientific research [137]. The issue extends beyond the mere absence of a universally accepted biomarker or neuromarker; it involves the intricate task of authentically characterizing an inherently idiosyncratic event that exhibits not only interindividual variations but also intraindividual differences influenced by contextual factors. Philosophical perspectives in the realm of mind theory have ventured towards eliminativism in this regard. Yet, scientific inquiry necessitates a form of generalization that can achieve equilibrium, acknowledging the commonalities we discern while still honoring the unique individual variances [137].

In conclusion, the concept of a specific “Pain Neuromatrix” has been both defended and challenged over recent years. This stark divide showcases the intricate nature of pain and how it is processed by our neural networks. Modern research suggests that the perception of pain is likely not tied to a single neural network but instead emerges from a multifaceted network responding to important sensory stimuli. This view emphasizes the key role of sensory stimuli in the pain experience. Additionally, innovative models like the Bayesian Model propose a novel perspective on pain, framing it as a probabilistic and inferential process, where perception is shaped not only by present sensory information but also by past experiences. The study of cortical processing and pain shines a light on the complex interaction between pain and consciousness. This understanding is crucial for guiding future research and holds significant potential for enhancing the clinical management of chronic pain.

## 7. Summary

**Figure 1:**
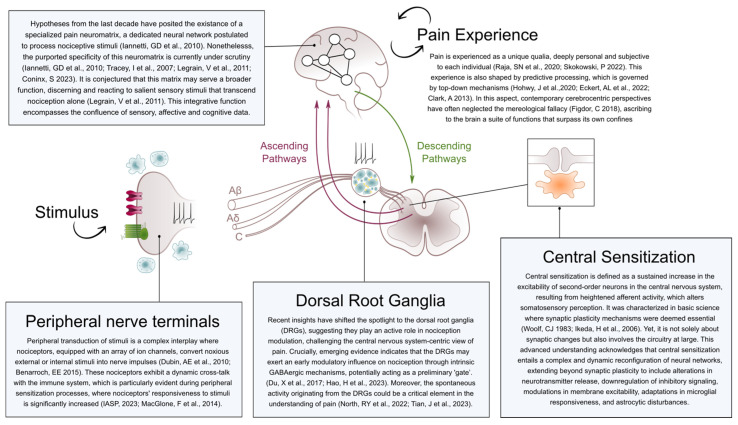
Schematic overview of the principal elements addressed in this narrative review.

The investigation into pain has uncovered intricacies that challenge conventional and reductionist views we might have held to date. With the advent of neurosciences, we might have fallen into misconceptions that have permeated various fields. In this regard, pain was conceived as a product of the brain, thereby succumbing to the mereological fallacy—a fallacy that should be avoided when studying complex phenomena dependent on emergent properties arising from interactions within system components (Figure 1).

Consequently, there is a prevalent confusion between pain’s multidimensionality (qualia or the individual experience of pain) with the neurophysiology of nociception. However, caution is imperative. While they are not identical concepts, they are intricately related; it is challenging to evidence pain without nociception.

Align with this, to explain the perpetuation of chronic pain, the focus has been on underlying central mechanisms. This trend has often overshadowed the crucial role of the peripheral nervous system, where substantial evidence suggests its active involvement in pain chronification. This goes beyond the communication between the peripheral and central nervous systems but also the cross-talk between the nervous system and other systems. This perspective challenges views that overemphasize the brain as the primary pain generator, underscoring the importance of peripheral contributions.

Similarly, central sensitization has been proposed as a mechanism responsible for pain chronification, characterized by an increase in the hyperexcitability of the second neuron in the dorsal horn. Yet, its significance in some domains has become an umbrella term to justify vague clinical contexts. In clinical taxonomy, it is crucial to critically assess its role as the primary etiopathogenesis of clinical presentations. Central sensitization has been described in basic research and, therefore, is contentious when generalized to clinical contexts. Presently, there is no evidence demonstrating central sensitization as described by Woolf in human subjects, and current evaluation tools for humans, such as central sensitization questionnaires, show controversial correlations with sensory aspects. On the other hand, quantitative sensory testing could provide patient sensory data that might suggest, or not, the presence of central sensitization, as these are not a direct measure of the neurophysiological phenomena. Thus, it seems more appropriate to suggest that it enables the evaluation of nociceptive signal modulation rather than directly indicating central sensitization itself. Therefore, it is vital to adopt more suitable concepts, given the complex nature of widespread pain pathologies.

Finally, in the past decade, pain awareness has been attributed to a possible specific neuromatrix composed of various brain regions. The specificity of this “Pain Neuromatrix” has been debated, highlighting the inherent complexity of pain. 

Recent research against this theory suggests that rather than processing pain-associated information, it processes salient sensory information significant to the organism without an evident specificity. Contemporary theories, in line with consciousness models, propose pain from a Bayesian Model that perceives pain consciousness from a probabilistic and inferential approach, influenced by both current sensory information and past experiences. In this light, we cannot detach pain experience from consciousness research. Comprehending pain as an experience necessitates a deep understanding of the complexities inherent in consciousness.

In summary, the study of pain and nociception is an expansive and multifaceted field that necessitates an approach recognizing the interplay among diverse systems. This understanding is paramount for guiding future research and enhancing the clinical management of chronic pain.

## Data Availability

Data sharing not applicable.
